# Loss of function of FIP200 in human pluripotent stem cell-derived neurons leads to axonal pathology and hyperactivity

**DOI:** 10.1038/s41398-023-02432-3

**Published:** 2023-05-03

**Authors:** Jianbin Wen, Andreas Zellner, Nils Christian Braun, Thomas Bajaj, Nils Christian Gassen, Michael Peitz, Oliver Brüstle

**Affiliations:** 1 Institute of Reconstructive Neurobiology, University of Bonn Medical Faculty & University Hospital Bonn, Bonn, Germany; 2 Department of Physiology, School of Basic Medicine and Tongji Medical College, Huazhong University of Science and Technology, Wuhan, China; 3 Research Group Neurohomeostasis, Clinic and Polyclinic for Psychiatry and Psychotherapy, University of Bonn Medical Faculty & University Hospital Bonn, Bonn, Germany; 4 Cell Programming Core Facility, University of Bonn Medical Faculty, Bonn, Germany

**Keywords:** Stem cells, Molecular neuroscience

## Abstract

FIP200 plays important roles in homeostatic processes such as autophagy and signaling pathways such as focal adhesion kinase (FAK) signaling. Furthermore, genetic studies suggest an association of *FIP200* mutations with psychiatric disorders. However, its potential connections to psychiatric disorders and specific roles in human neurons are not clear. We set out to establish a human-specific model to study the functional consequences of neuronal FIP200 deficiency. To this end, we generated two independent sets of isogenic human pluripotent stem cell lines with homozygous FIP200^KO^ alleles, which were then used for the derivation of glutamatergic neurons via forced expression of NGN2. FIP200^KO^ neurons exhibited pathological axonal swellings, showed autophagy deficiency, and subsequently elevated p62 protein levels. Moreover, monitoring the electrophysiological activity of neuronal cultures on multi-electrode arrays revealed that FIP200^KO^ resulted in a hyperactive network. This hyperactivity could be abolished by glutamatergic receptor antagonist CNQX, suggesting a strengthened glutamatergic synaptic activation in FIP200^KO^ neurons. Furthermore, cell surface proteomic analysis revealed metabolic dysregulation and abnormal cell adhesion-related processes in FIP200^KO^ neurons. Interestingly, an ULK1/2-specific autophagy inhibitor could recapitulate axonal swellings and hyperactivity in wild-type neurons, whereas inhibition of FAK signaling was able to normalize the hyperactivity of FIP200^KO^ neurons. These results suggest that impaired autophagy and presumably also disinhibition of FAK can contribute to the hyperactivity of FIP200^KO^ neuronal networks, whereas pathological axonal swellings are primarily due to autophagy deficiency. Taken together, our study reveals the consequences of FIP200 deficiency in induced human glutamatergic neurons, which might, in the end, help to understand cellular pathomechanisms contributing to neuropsychiatric conditions.

## Introduction

Multiple studies have associated *FIP200* (FAK family kinase-interacting protein of 200 kDa, aka *RB1CC1*) with psychiatric disorders. Duplications affecting the *FIP200* gene were reported in children with developmental delay [1] and patients suffering from autism [2]. A de novo mutation causing a frameshift in the *FIP200* gene was also reported in a schizophrenia patient [3]. Importantly, a large cohort study identified duplication of *FIP200* as the most significantly over-represented risk factor for the development of schizophrenia [4]. A schizophrenia patient carrying a de novo complete duplication resulting in overexpression of FIP200 was also reported recently [5]. These observations point to a significant role of FIP200 in the pathogenesis of psychiatric disorders and call for studies into the role of this gene in the human central nervous system.

The human *FIP200* gene is localized on chromosome 8q11, encoding a protein with 1594 amino acid residues. Early studies suggested that FIP200 binds to the kinase domain of FAK, and inhibits its kinase activity [6, 7]. Later studies revealed that FIP200 also functions in macroautophagy (hereafter referred to as autophagy) [8, 9]. It forms dimers and contributes to the organization of the ULK1 complex with its N-terminal domain, while its C-terminus forms a “claw” structure to capture receptor-labeled cargos for the autophagy machinery [10–12].

Deletion of FIP200 in mice leads to embryonic death at mid/late gestation associated with heart failure and liver degeneration [13]. Mice with neural-specific deletion of FIP200 show cerebellar degeneration accompanied by progressive neuronal loss, spongiosis, and neurite degeneration [14]. FIP200 loss of function also impairs maintenance and differentiation of postnatal neural stem cells within the subventricular zone, a phenotype that can be attributed to intracellular radical oxygen species dysregulation and microglia activation [15, 16]. Deletion of FIP200 in Bestrophin 1-positive cells leads to a loss of photoreceptors and the degeneration of the retinal pigment epithelium [17]. While these studies point to broad biophysiological implications associated with FIP200 loss of function in the nervous system, the roles of FIP200 in neuronal development and function remained largely elusive. However, such data are a prerequisite for deciphering the molecular mechanisms by which FIP200 variants contribute to the pathogenesis of psychiatric disorders such as schizophrenia.

Here we set out to study the function of FIP200 in forebrain neurons derived from human pluripotent stem cells (hPSCs). We used CRISPR-Cas9-based genome editing to introduce FIP200 loss of function mutations in hPSCs, and employed a transcription factor-based “forward-programming” approach to generate induced glutamatergic neurons (iGlutNs). We report the morphological and electrophysiological consequences of FIP200 loss of function in these neurons, and explore how autophagy- and FAK-dependent pathways might mediate these alterations. We expect these results to provide an important basis for further deciphering the role of FIP200 mutations in the pathogenesis of psychiatric disorders.

## Materials and methods

### Human pluripotent stem cell culture and genome editing

Two hPSC lines were used in the present study: an induced pluripotent stem cell line generated in-house from skin fibroblasts of a healthy Caucasian male (iLB-C14m-s11; abbreviated C14, registered at hPSCreg® as UKBi017-A (https://hpscreg.eu/cell-line/UKBi017-A)), and the male human embryonic stem cell line WA01 (aka H1) obtained from the WiCell Research Institute (Wisconsin, USA). hPSCs were cultured in plates coated with Matrigel (1:100 dilution, Corning, 354230) in an hPSC maintenance medium (StemMACS iPS-Brew XF, Miltenyi Biotec, 130104368). A mycoplasma test was performed every 2 weeks. For genome editing, hPSCs were prepared to reach 80% confluency, dissociated with Accutase (Thermo Fisher, A11105), and 3 × 10^4^ cells were resuspended in 20 µL nucleofection solution (P3 Primary Cell 4D Nucleofector kit, Lonza, V4XP-3012) containing RNPs composed of the Alt-R HiFi CRISPR-Cas9 nuclease (IDT, 1081059) and the cr::tracrRNA (Hs.Cas9.RB1CC1.1.AB: CAAGATTGCTATTCAACACC; Alt-R® CRISPR-Cas9 tracrRNA, IDT, 1072532). Nucleofection was performed with an Amaxa 4D Nucleofector (Lonza) using the program CM150. Single-cell-derived colonies were manually isolated and expanded. For genotyping analyses, a PCR amplicon was generated using GoTaq® DNA Polymerase (Promega, M3001) with primers: Fwd, 5′-GGGGAAGGTTTTAGAGTGAAATT-3′ and Rev, 5′-GAAGGCAATGTGCACGCTCA-3′. PCR products were then send for Sanger sequencing (Microsynth Seqlab) as well as amplicon sequencing (GENEWIZ, Inc.).

### Immunocytochemistry

Cultures maintained in Matrigel-coated 96-well-plates were fixed with 4% PFA (Sigma-Aldrich, 100496) for 12 min, blocked with PBS containing 10% FBS (Thermo Fisher, 10500064) and 0.1% Triton X-100 (Sigma-Aldrich, T8787) for 1 h at room temperature, and then incubated with primary antibodies (anti-SOX2 mouse, 1:1000, R&D Systems MAB2018; anti-OCT4 rabbit, 1:500, Santa Cruz sc-9081; anti-GABA rabbit, 1:1000, Sigma A2052; and anti-TUBB3 1:250 chicken, Millipore AB9354) in blocking buffer at 4 °C overnight. The cultures were then washed three times with PBS and incubated with secondary antibodies (Alexa 488 goat anti-mouse IgG, 1:1000, Alexa 555 goat anti-chicken IgG, 1:500, and Alexa 647 goat anti-rabbit IgG, 1:500, all from Thermo Fisher) in blocking buffer at room temperature for 1 h. The solution was replaced with 1 µg/mL DAPI (Thermo Fisher, D1306) in PBS for 3 min and then washed once with PBS. Imaging was then conducted with an IN Cell Analyzer 2200 (GE Healthcare) and analyzed with ImageJ [18].

### Generation and cultivation of iGlutNs

The iGlutNs were generated as previously described in detail [19]. To that end, doxycycline was applied for the induction of NGN2 expression at the hPSC stage. After eight days of induction, the resulting iGlutNs were dissociated and cryopreserved as batches. For morphological analysis, iGlutNs were thawed and seeded on a confluent layer of inactivated mouse astrocytes at a density of 5 × 10^4^ cells/well in neuronal medium (Neurobasal Medium (Thermo Fisher Scientific, 21103-049), 2% B27 supplement (Thermo Fisher Scientific, 17504-044), 1% GlutaMax (Thermo Fisher Scientific, 35050-38), and 10 ng/mL BDNF (Cell Guidance Systems, GFH1). For electrophysiological recordings, iGlutNs were seeded in Matrigel-coated 24-well plates with micro-electrode arrays at a density of 3 × 10^4^ cells/well, and 3 × 10^4^ mouse astrocytes were added the next day. The co-cultures were maintained in neuronal medium supplemented with 1 µg/ml doxycycline and 0.5% FBS, and a half medium change was performed twice per week. For immunoblotting analyses, iGlutNs were seeded in Matrigel-coated six-well-plates at a density of 1 × 10^6^ cells/well, and cultured with astrocyte-conditioned neuronal medium supplemented with 1 µg/ml doxycycline while changing half of the medium twice per week. For long-term treatment with the FAK inhibitor PF573228 (Tocris 3239) or the ULK1 inhibitor MRT68921 (Tocris 5780), compounds were added immediately after thawing of the neuronal cultures and replenished with each media change every 3–4 days.

### Protein immunoblot

Cultures were washed once with ice-cold PBS, scratched off in cold PBS, and centrifuged. The pellet was resuspended in cold RIPA buffer (Sigma-Aldrich, R0278) supplemented with 1:100 HALT protease and phosphatase inhibitor (Thermo Fisher, 78442). The lysate was incubated on ice for 30 min while vortexing every 5 min and then centrifuged for 30 min with 16,100×*g* at 4 °C. The protein concentration of the supernatant was determined using the Pierce BCA Protein Assay kit (Thermo Fisher, 23225). About 30 µg protein samples were mixed with NuPAGE™ LDS Sample Buffer (Thermo Fisher, 84788) and loaded onto a 10% Bis-Tris gel (Thermo Fisher, NP0315BOX) for electrophoresis and then electroblotted onto a methanol-activated PVDF membrane (Bio-Rad Laboratories, 1620177). Afterwards, the membrane was blocked for 1 h at room temperature in TBS-T buffer (25 mM Tris-Base, 150 mM NaCl, and 0.1% Tween-20) containing 5% milk powder (Carl Roth, T145), and incubated with the primary antibody diluted in blocking buffer (anti-FIP200, 1:1000, Sigma-Aldrich SAB4200135; anti-GAPDH 1:1000, Santa Cruz sc47724; anti-p62 1:1000, Abnova H00008878-M01; anti-Ubiquitin 1:200, Santa Cruz sc-8017; anti-PSD95, 1:1000, Thermo Fisher MA1064; anti-Synapsin1, 1:1000, Synaptic Systems 106103) overnight at 4 °C. The membrane was then washed 3 times with TBS-T buffer and incubated with HRP-conjugated secondary antibodies (anti-mouse IgG, 1:1000, Cell signaling 7074; and anti-rabbit IgG, 1:1000, Cell signaling 7076) in blocking solution for 1 h at room temperature. Luminata™ Classico/Crescendo/Forte (Merck Millipore) were used for chemiluminescence detection in a Chemidoc XRS system (Bio-Rad Laboratories). Signal quantification was performed using the Image Lab software (Bio-Rad Laboratories).

### Transfection of reporter constructs

For each well of a 96-well plate, 100 ng plasmid and 0.3 µL Fugene reagent (Promega, E2311) were mixed with 10 µL neuronal medium, incubated at room temperature for 10 min, and added to each well. Two reporter plasmids were used in the present study. The autophagy reporter plasmid expressing rat LC3 fused to mRFP and EGFP under the control of a CMV promotor (ptfLC3), was generated by Tamotsu Yoshimori et al. [20], and obtained via Addgene (#21074). To label individual neurons in dense cultures, a cassette expressing humanized renilla reniformis green fluorescent protein (hrGFP) under the control of Doublecortin (DCX) promoter was cloned into a pMA vector. The hrGFP was amplified from a plasmid constructed by Su-Chun Zhang et al. [21] and obtained from Addgene (#52344). The DCX promoter was amplified from a lentiviral construct created by Ladewig et al. [22], and the pMA vector was obtained from GeneArt (GeneArt AG).

### Live cell imaging and analysis

Live fluorescence imaging was performed with a Leica live cell imaging system (Leica Microsystems) 7–10 days after the plasmid transfection. The plate was kept in a chamber with a temperature at 37 °C and CO_2_ at 5%. Sholl analysis was performed using the Sholl Analysis plug-in for ImageJ [23]. Axonal swellings as well as the number of autophagosomes and autolysosomes were counted manually.

### Multi-electrode array recordings

For all electrophysiological recordings, cultures were maintained in 24-well MEA plates (M384-tMEA-24W, Axion BioSystems). Each well contains 16 electrodes arranged in a 4 × 4 grid covering an area of 1.1 mm × 1.1 mm. The electrodes have a diameter of 50 µm and a center-to-center distance of 350 µm. A Maestro MEA EDGE system (Axion BioSystems) was used for electrophysiological data acquisition. For recording, the plate was loaded into a chamber with the temperature maintained at 37 °C and CO_2_ at 5%. Raw data sets were recorded with the sampling rate at 12.5 kHz and a bandpass between 0.1–2000 Hz. Online spike detection was performed with a threshold of six times the standard deviation. For the recording of spontaneous activities, the plate was first loaded into the chamber and equilibrated for 20–30 min, and then recorded for 10 min. For acute pharmacological stimulation experiments, compounds of 20x working concentration were added into cultures and recordings were performed after 10 min of equilibration. After treatment, cultures were washed three times with fresh medium and recorded again after 20–30 min of equilibration. Recorded data sets were processed using the Navigator software (Axion BioSystems).

### Membrane protein biotinylation and quantitative mass spectrometry

Astrocyte-neuronal co-cultures were washed three times with ice-cold PBS and biotinylated with 1 mg/ml sulfo-NHS-SS-biotin (Thermo Fisher, 21331) in cold PBS for 45 min. Following the protein extraction steps described above, the supernatant was then used for streptavidin precipitation employing magnetic streptavidin beads (Thermo Fisher, 88817). After rotating overnight at 4 °C, beads were washed with RIPA buffer and the final pellet was subjected to mass spectrometry. For mass spectrometry, PBS-washed beads were digested overnight at 37 °C with 1 μg trypsin (Promega, V5111). Using the nanoelectrospray interface, resulting peptides were sprayed into a timsTOF Pro mass spectrometer (Bruker Daltonics). Raw data were processed using the MaxQuant computational platform (v2.0.1.0). The peak list was searched against the canonical Uniprot database of *Homo sapiens* and *Mus musculus* (20370 and 17048 entries, October 2020). Proteins were quantified across samples using the label-free quantification algorithm in MaxQuant as label-free quantification (LFQ) intensities. Gene ontology analysis of cellular components was performed using g:Profiler [24]. LFQ intensities of each mapped protein were first normalized by subtracting the median intensity, then compared between genotypes with Student’s *t*-test and corrected with the false discovery rate (FDR) method. *T* values from the *t*-test of each protein were also used for a gene set enrichment analysis (GSEA) using WebGestalt 2019. The GSEA was performed using enrichment category “gene ontology Biological Process noRedundant” and the significance level was set to FDR <0.05.

### Statistical analyses

Statistical analysis was performed using MATLAB. Unless otherwise noted, data were subjected to Kolmogorov–Smirnov test for normal distribution before being analyzed with N-way analysis of variance (ANOVA) with unequal variances, and only the main effect of genotype (control vs. KO) was reported; paired comparisons were then corrected by using the Tukey–Kramer method.

## Results

### Establishment of isogenic FIP200^KO^ hPSC lines and forward-programmed neurons

**Fig. 1:**
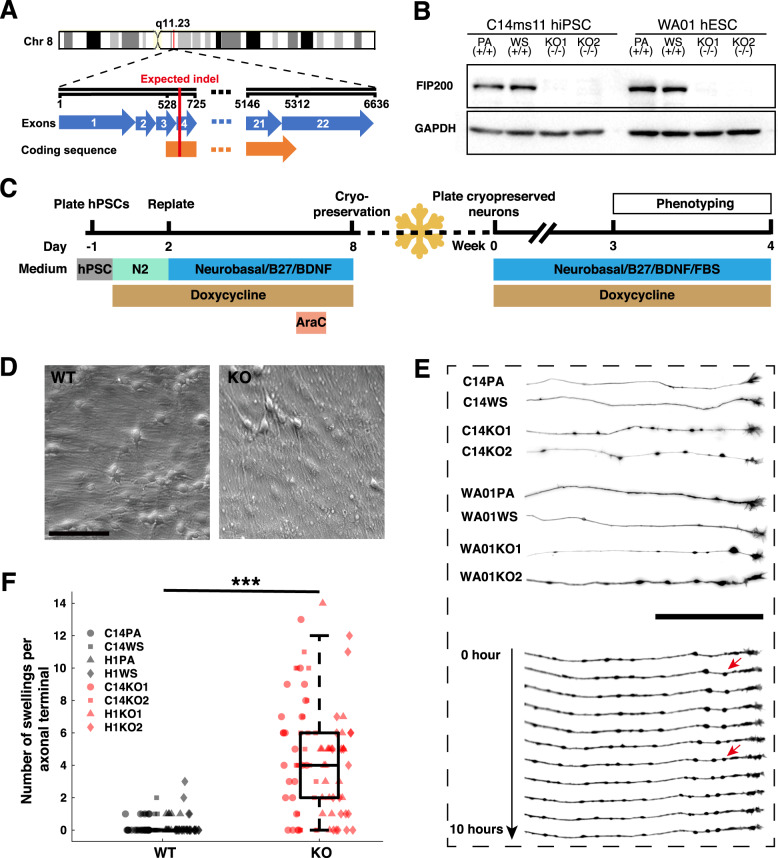
Establishment of FIP200^KO^ human iGlutNs and morphological assessment. **A** Genomic localization of *FIP200* and CRISPR-Cas9-mediated editing approach to create indels in exon 4. **B** Protein expression of FIP200 in obtained hPSC lines assessed by western blot. **C** Schematic workflow of the iGlutN generation protocol, neuronal maturation, and phenotyping timeframe. **D** Example pictures from live phase contrast microscopy of wild type and FIP200^KO^ iGlutN cultures during week 4 of maturation on astrocytes. **E** Upper panel: Representative inverted live fluorescence pictures of axonal terminals from wild type and FIP200^KO^ iGlutNs. Lower panel: Live time-lapse microscopy of an axonal terminal of a FIP200^KO^ neuron imaged hourly up to 10 h. Red arrows indicate newly formed swellings. **F** Quantification of axonal swellings in wild type and FIP200^KO^ iGlutN cultures. For each cell line, >20 axons from three independent experiments were analyzed. Scale bar, 100 µm. Two-way ANOVA with Tukey–Kramer correction for post hoc paired comparisons. PA parental line, WS wild-type subclone, KO knock-out line. ***p < 0.001.

Two hPSC lines (hiPSC C14 and hESC WA01) carrying a doxycycline-inducible human NGN2 expression cassette in both alleles of the AAVS1 “genomic safe harbor” locus established previously were used in the present study [19]. Overexpression of NGN2 in hPSCs has previously been shown to efficiently induce forebrain excitatory neurons [25]. Both cell lines were subjected to CRISPR-Cas9-mediated genome editing targeting exon 4 of *FIP200* (Fig. 1A). From each background, we chose two homozygous knockout lines (KO1 and KO2, Supplementary Table S1), and one unedited wild-type subclone (WS) as control, respectively. Moreover, the parental lines (PA) were used as additional controls. This yielded two sets of isogenic hPSC lines (i.e., C14-PA (+/+), C14-WS (+/+), C14-KO1 (−/−), C14-KO2 (−/−), and WA01-PA (+/+), WA01-WS (+/+), WA01-KO1 (−/−), WA01-KO2 (−/–)), which were used in all downstream experiments in parallel. For all obtained cell lines, deep sequencing was performed to exclude the presence of rare additional alleles due to possible low-grade mosaicism (Supplementary Fig. S1). In addition, SNP-based virtual karyotyping was performed to verify the genome integrity (Supplementary Fig. S2). The absence of FIP200 protein was verified by western blot (Fig. 1B). Robust expression of pluripotency markers, OCT4 and SOX2, was confirmed by immunocytochemistry (Supplementary Fig. S3A), indicating that neither the insertion of the doxycycline-inducible NGN2 expression cassette into the AAVS1 locus nor the loss of function of FIP200 had severe effects on pluripotency.

### Axonal aberrations in FIP200^KO^ iGlutNs

FIP200^KO^ and control hPSC lines were then subjected to a forward-programming protocol for the generation of forebrain excitatory neurons as previously described [19]. No overt morphological differences were observed between the different genotypes during this early neuronal induction process (Supplementary Fig. S3B). For subsequent phenotyping assays, we thawed cryopreserved iGlutNs and matured them for 3–4 weeks under neurotrophic conditions (for an overview of the differentiation and maturation protocol, see Fig. 1C). After a post-thaw maturation time of 3 weeks, we observed multiple “swelling” structures in FIP200^KO^ iGlutNs cultures as compared to wild-type cultures (Fig. 1D). Morphological assessment of single neurons in these cultures with live fluorescence imaging indicated that swelling structures are predominantly present along axons, especially close to the distal axonal terminal. Frequently, swellings formed just behind the axonal growth cone (Fig. 1E). Compared to wild-type controls, the number of swellings per axonal terminal was significantly higher in FIP200^KO^ iGlutNs (median count 4 vs. 0, *F*(1,187) = 170.4, *p* < 0.0001, Fig. 1F). Nevertheless, loss of function of FIP200 appeared not to affect dendrite or axon outgrowth, as no significant difference could be observed in dendrite complexity as quantified by Sholl analysis (Supplementary Fig. S4A, B), or in the speed of axon growth (median speed 2.7 vs. 2.1 μm/h, *F*(1,48) = 3.06, *p* = 0.087, Supplementary Fig. S4C, D) between cultures of different genotypes.

### FIP200^KO^ iGlutNs show autophagy deficiency

**Fig. 2:**
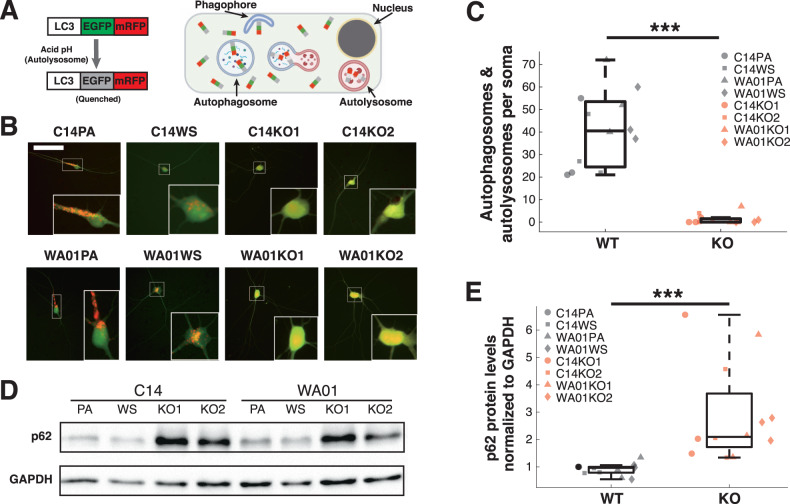
FIP200 loss of function leads to autophagy deficiency in iGlutNs. **A** Schematic illustration of the autophagy reporter (ptfLC3) used in the present study. EGFP- and RFP-tagged LC3 is recruited to autophagosomes, and after fusion of autophagosomes and lysosomes, only eGFP is quenched, resulting in red fluorescent autolysosomes. **B** Representative live fluorescence microscopy pictures of iGlutNs 7 days after transfection with the autophagy reporter. Scale bar, 300 µm. **C** Autophagosome and autolysosome vesicle quantification in wild type and FIP200^KO^ iGlutNs (*n* = 3). **D**, **E** Example western blot (**D**) and immunoblot quantification (**E**) for assessing p62 levels in wild type and FIP200^KO^ iGlutN culture lysates (*n* = 3). Samples were collected during week 4 of maturation. Two-way ANOVA with Tukey–Kramer correction for post hoc paired comparisons. PA parental line, WS wild-type subclone, WT wild-type line, KO knock-out line. ***p < 0.001.

To assess the effect of FIP200 loss of function on autophagy, we transfected the cultures with a plasmid expressing mRFP-GFP tandem fluorescent-tagged LC3 (pftLC3), the gold standard for measuring the autophagic flux in cells [20]. In transfected cells, mRFP and GFP are equally distributed before and during the early stages of autophagy. However, after the fusion of autophagosome and lysosome, GFP is quenched in acidic and degradative conditions, whereas mRFP-LC3 is not; therefore, the autolysosomes are marked with red fluorescence (Fig. 2A). We observed a large number of red-colored vesicles in wild-type iGlutNs, predominantly near and within the soma, but very few in FIP200^KO^ iGlutNs (Fig. 2B), and the difference in the combined numbers of autophagosome and autolysosome vesicles, as an indicator of autophagosome formation, was highly significant (*F*(1,23) = 105.52, *p* < 0.0001, Fig. 2C and Supplementary Fig. S5). Autophagy deficiency also leaves the autophagosomal receptor p62 accumulated in the cytoplasm. This was also confirmed in FIP200^KO^ iGlutNs, which showed significantly higher p62 levels than wild-type controls, as revealed by western blot analysis (*F*(1,23) = 22.4, *p* = 0.00014, Fig. 2D, E). By contrast, ubiquitin seemed not to accumulate in FIP200^KO^ iGlutNs (*F*(1,23) = 0, *p* = 0.96, Supplementary Fig. S6), suggesting that proteasome activity was not attenuated in FIP200-deficient neurons. Together, these results confirm that the loss of function of FIP200 leads to the impairment of autophagy.

### FIP200-deficient human iGlutN networks are hyperactive

**Fig. 3:**
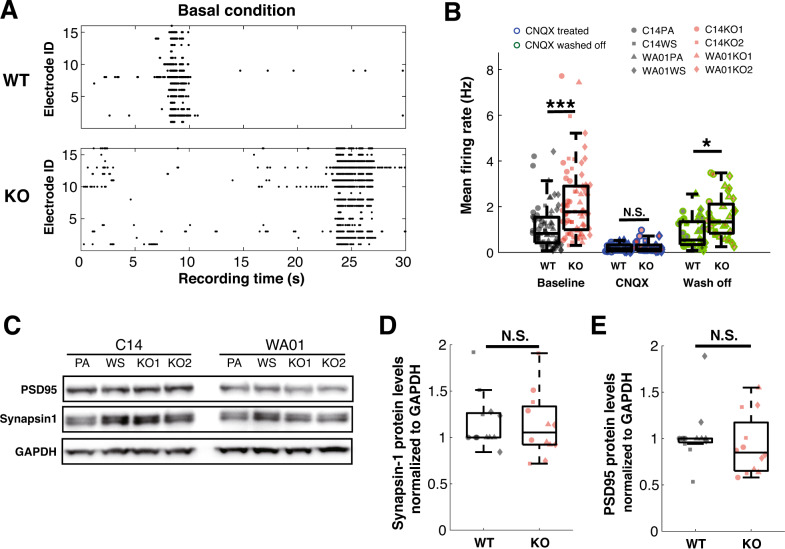
Electrophysiological analysis of the wild-type and FIP200^KO^ iGlutN cultures using MEAs. **A** Example raster plots of spike activities recorded from wild-type and FIP200^KO^ iGlutN cultures in basal conditions. **B** Quantification of the spiking activity recorded from wild-type and FIP200^KO^ iGlutN cultures under baseline condition, treated with 10 µM CNQX, and after washing off CNQX. For each cell line, nine cultures from three independent experiments were analyzed. Three-way ANOVA with Tukey–Kramer correction for post hoc paired comparisons. **C**–**E** Quantification of the presynaptic marker Synapsin1 (**C**, **D**) and the post-synaptic marker PSD95 (**C**, **E**) by western blot analysis (*n* = 3; protein levels were normalized to GAPDH). All experiments were performed during week 4 of maturation on mouse astrocytes. PA parental line, WS wild-type subclone, WT wild-type line, KO knock-out line. Two-way ANOVA with Tukey–Kramer correction for post hoc paired comparisons. *p < 0.05, ***p < 0.001, NS not significant.

We next set out to explore possible electrophysiological changes that could result from FIP200 deficiency in human iGlutNs. Upon maturation on mouse astrocytes for three weeks, FIP200^KO^ iGlutN cultures exhibited more spiking activity than wild-type controls (median of mean firing rate: 1.77 vs. 0.83 Hz, *p* < 0.0001, Fig. 3A, B). This elevated activity appears to be due to enhanced network activity rather than an increased spontaneous spiking activity, as after the application of CNQX, an α-amino-3-hydroxy-5-methyl-4-isoxazolepropionic acid (AMPA) and kainate receptor antagonist, no significant difference of spiking activity between genotypes could be observed (median of mean firing rate: 0.11 vs. 0.14 Hz, *p* = 0.891, Fig. 3B). Increased spiking activity in FIP200^KO^ iGlutNs re-appeared 30 min after washing off CNQX (median of mean firing rate: 1.62 vs. 1.15 Hz, *p* = 0.0387, Fig. 3B). Since NGN2-induced iPSC-derived neuronal cultures can contain small numbers of GABAergic neurons, we also assessed whether the differences in spiking activity could be due to different ratios of GABAergic neurons in FIP200^KO^ and control cultures. Quantification of GABA-positive neurons did not reveal significant differences between control and FIP200-deficient cultures (*F*(1,99) = 0.96, *p* = 0.3286, Supplementary Fig. S7A). Moreover, the application of bicuculline, a GABA-A receptor antagonist, did not elicit a significant change in network activities in cultures of either genotype (*p* = 0.28 for WT cultures, *p* = 0.34 for FIP200^KO^ cultures, paired *t*-test, Supplementary Fig. S7B), and also the scale of change was not significantly different between the two genotypes (median fold change of mean firing rate: 0.1 vs. 0.06, *F*(1,23) = 1.43, *p* = 0.241, Supplementary Fig. S7C). Thus, the observed hyperactivity appears to be due to increased glutamatergic synaptic activation. We then wondered whether the differences might be due to differences in the synaptic build-up. Quantification of pre- and post-synaptic markers synapsin1 and PSD95 with western blot failed to reveal significant differences (*F*(1,23) = 0.09, *p* = 0.764 for synapsin1, and *F*(1,23) = 0.64, *p* = 0.432 for PSD95, respectively; Fig. 3C–E). Therefore, the stronger glutamatergic excitation in FIP200^KO^ iGlutNs is unlikely due to an excessive synaptic build-up.

### FIP200^KO^ iGlutNs show shifted metabolic processes and dysregulation in cell adhesion-related processes

**Fig. 4:**
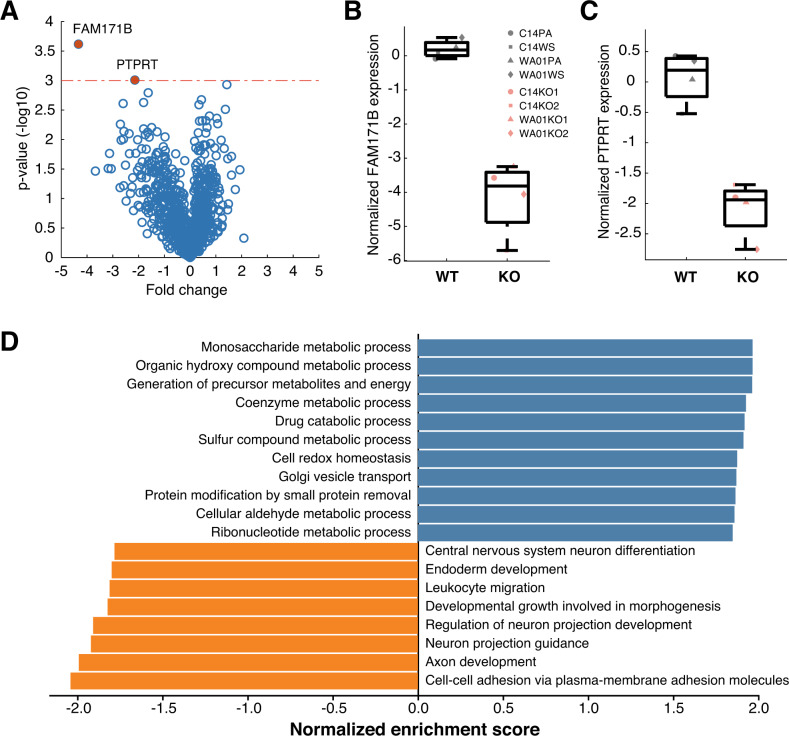
Membrane proteomic analysis of FIP200^KO^ and isogenic control iGlutN cultures. **A** Volcano plot of all 1043 identified human proteins. Cut off by FDR *q* < 0.05. **B**, **C** FAM171B and PTPRT are significantly downregulated in FIP200KO iGlutN cultures compared to controls. **D** Gene set enrichment analysis of membrane proteome analysis revealing biological processes significantly up- or downregulated in FIP200^KO^ iGlutN vs. isogenic wild-type cultures. Data were based on cultures matured for 3 weeks (one sample for each of the eight cell lines).

Proteins that directly support the electrophysiological machinery of neurons mostly reside on the membrane. To further investigate which component mediated the observed hyperactivity in FIP200^KO^ iGlutN cultures, we performed cell surface proteomic analyses with human iGlutN cultures matured on mouse astrocytes for 3 weeks. Among the 2007 proteins that were detected in all samples, 1043 could be mapped to the human proteome (Supplementary Table S2). In order to further stratify the data and to assess the degree of membrane specificity of our proteome analysis, a gene ontology (GO) analysis relating to cellular compartments was performed. 951 of 1043 identified human proteins could be assigned to three locations, i.e., extracellular compartment, plasma membrane, and cytoplasm (Supplementary Table S3). The results revealed that the majority of these proteins are associated with the plasma membrane (488, 51%) or the extracellular compartment (193, 20%). However, among the identified proteins involved in the glutamatergic transmission, i.e., glutamate receptors (GRIA1-4, GRID1, and GRM3) and glutamate carboxypeptidase 2 (FOLH1), we did not observe significantly different expression levels in FIP200^KO^ and control neurons. Further proteins that are essential for synaptogenesis and function, including NRXN2, NRXN3, GABBR1, GABBR2, GABRB3, and VPS13A, also showed no significant difference in expression. It thus remains unclear which mechanisms mediate the hyperactivity in FIP200^KO^ neuronal cultures. Nevertheless, two proteins did show significantly lower expression in FIP200^KO^ samples than in controls after correction for multiple testing: FAM171B (family with sequence similarity 171 member B) and PTPRT (receptor-type tyrosine-protein phosphatase T) (Fig. 4A–C). FAM171B is a single-pass type I membrane protein containing a polyglutamine (polyQ) stretch [26]. It is widely expressed in the brain and can be recruited into polyQ aggregates and thus contribute to the development of polyQ diseases [27]. While it is unclear how FAM171B might be related to FIP200, PTPRT could be linked to FIP200 via FAK pathways. PTPRT is exclusively expressed in the brain and regulates synapse formation through interaction with cell adhesion molecules [28]. Its reduction on the neuronal membrane, where FAK is presumably disinhibited in the absence of FIP200 [6], suggests a dysregulation of protein tyrosine kinase phosphatases in processes related to cell adhesion. This hypothesis was supported by a subsequent GO analysis of the biological process. It revealed that the most significantly downregulated GO term in FIP200^KO^ neurons is cell-cell adhesion, with other downregulated terms also being related to cell adhesion, e.g., axon development, neuron projection, morphogenesis, and migration. Interestingly, GO terms that are significantly positively enriched also point to the other key function of FIP200, i.e., the regulation of autophagy and, thus, metabolic control. For example, GO terms significantly enriched in FIP200^KO^ neurons included “generation of precursor metabolites” in many metabolic processes, as well as “cell redox homeostasis” and proteolysis (Fig. 4D and Supplementary Table S4). These results indicate that FIP200 impacts cellular processes via autophagy- and possibly FAK-related functions in human iGlutNs.

### Impaired autophagy and altered FAK signaling may contribute to the hyperactivity of FIP200-deficient glutamatergic neurons

We then proceeded to further explore FIP200 deficiency by interfering with autophagy- and FAK-dependent pathways in iGlutN cultures to further support our hypothesis. As FIP200 functions as a member of the ULK1 complex in autophagy, we hypothesized that inhibition of the ULK1 complex in wild-type cultures might lead to similar autophagy deficiency as FIP200 loss of function. To explore this, we used MRT68921 (hereafter referred to as MRT), which is a potent inhibitor of ULK1 and ULK2, and can block autophagy at an early stage and cause accumulation of stalled early autophagosomal structures [29–31]. On the other hand, FIP200 has been shown to inhibit FAK [6, 7], a phenomenon which could explain the observed downregulation of PTPRT and cell adhesion-related proteins. We speculated that direct pharmacological interference with FAK pathways might reveal whether putative FAK disinhibition might contribute to the phenotypes observed in FIP200-deficient iGlutNs. To that end, we treated the cultures with the potent FAK inhibitor PF573228 (hereafter referred to as PF228) [32], and asked whether this could rescue the observed pathophenotypes in FIP200^KO^ neurons.

**Fig. 5:**
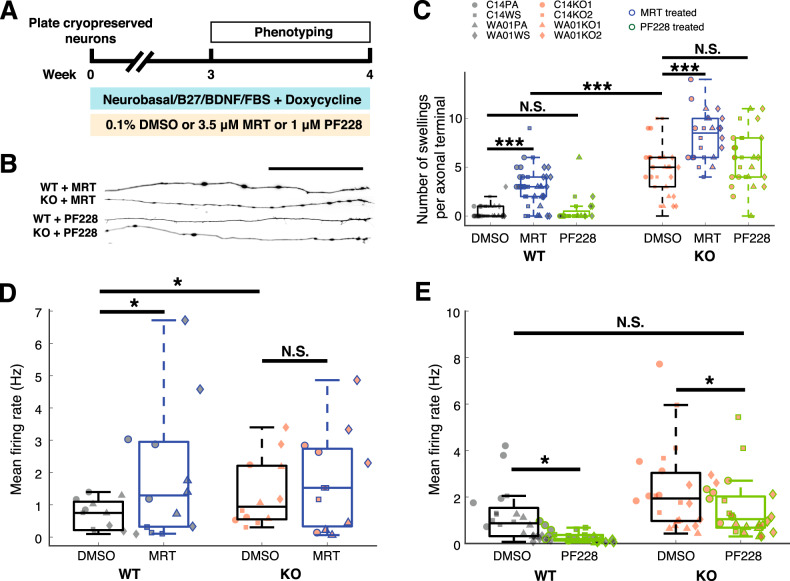
Pharmacological modulation of autophagy- and FAK-dependent functions in wild-type and FIP200^KO^ iGlutN cultures. **A** Related to Fig. 1C, 3.5 μM MRT (ULK1/2 inhibitor) or 1 μM PF228 (FAK inhibitor) were supplemented to iGlutN cultures until phenotypic characterization. **B** Example live fluorescence images of axonal terminals in iGlutN cultures supplemented with MRT or PF228. Scale bar, 100 µm. **C** Quantification of axonal swellings in iGlutN cultures under control conditions (0.1% DMSO) or in the presence of either 3.5 μM MRT or 1 μM PF228. For each condition, more than 20 axons from two independent experiments were analyzed. Three-way ANOVA with Tukey–Kramer correction for post hoc paired comparisons. **D** Quantification of spike activities recorded from iGlutN cultures in basal medium with 0.1% DMSO or treated with MRT. Hypothesis-driven one-tailed *t*-test was performed after a significant effect of MRT treatment was revealed by three-way ANOVA. **E** Quantification of spike activities recorded from iGlutN cultures in basal medium with 0.1% DMSO or treated with PF228. All recordings were performed during week 4 of maturation on mouse astrocytes. Three-way ANOVA with Tukey–Kramer correction for post hoc paired comparisons. PA parental line, WS wild-type subclone, WT wild-type line, KO knock-out line. *p < 0.05, ***p < 0.001, NS not significant.

MRT and PF228 were applied throughout the cultivation of post-thawed iGlutN cultures for 3–4 weeks (Fig. 5A). MRT arrested the initiation of autophagy in wild-type iGlutN cultures as revealed by the autophagy reporter, whereas PF228 did not, both as expected (Supplementary Fig. S8). MRT also led to axonal swellings in control iGlutN cultures (2.99 ± 0.28 vs. 0.07 ± 0.30, *p* < 0.0001; compared to non-MRT-treated cells), but not as prominent as those observed in FIP200^KO^ cultures in the control condition (2.99 ± 0.31 vs. 5.04 ± 0.31, *p* < 0.0001). Exposure to MRT further increased the number of axonal swellings in FIP200^KO^ cultures (5.04 ± 0.31 vs. 7.96 ± 0.33, *p* < 0.0001), indicating an additive effect. In contrast, PF228 treatment did not lead to significant differences in axonal swellings in wild-type (*p* = 0.568), or FIP200^KO^ (*p* = 0.568) iGlutN cultures (Fig. 5B, C). As for the electrophysiological properties, MRT treatment resulted in a marginal elevation of network activity in iGlutN cultures (*F*(1,47) = 4.19, *p* = 0.0466). Hypothesis-driven one-tailed *t*-test showed a marginal but significant increase of network activity in wild-type iGlutN cultures (mean firing rate: 1.755 ± 0.012 Hz vs. 1.352 ± 0.088 Hz, *p* = 0.0264), whereas in FIP200^KO^ cultures no significant effect could be observed (*p* = 0.2259, Fig. 5D). In comparison, PF228 treatment led to a decrease of spiking activity in both wild-type (mean firing rate: 0.393 ± 0.278 Hz vs. 1.418 ± 0.278 Hz, *p* = 0.0103) and FIP200^KO^ cultures (mean firing rate: 1.37 ± 0.278 Hz vs. 2.395 ± 0.278 Hz, *p* = 0.0159). Interestingly, PF228 treatment brought the activity in FIP200^KO^ iGlutN cultures to a level comparable to that of wild-type cultures without PF228 treatment (*p* = 0.9996, Fig. 5E), suggesting a successful rescue of the hyperactivity phenotype. Together, these results suggest that axonal swellings in FIP200^KO^ iGlutNs are induced by autophagy deficiency, whereas neuronal network hyperactivity may result from both impaired autophagy and disinhibited FAK signaling.

## Discussion

We found that loss of FIP200 impaired autophagy in induced human glutamatergic neurons, leading to p62 accumulation and axonal swellings. FIP200 deficiency also led to a hyperactive network characterized by increased glutamatergic synaptic activation. The proteomic analysis points to a dysregulated metabolism and abnormal processes related to cell adhesion in FIP200-deficient neurons. Pharmacological modulation with respective inhibitors suggests that both, impaired autophagy and the presumed disinhibition of FAK, contribute to the hyperactivity phenotype of FIP200-deficient neuronal networks, while the pathological axonal swellings are mainly due to the impairment of autophagy.

Axonal swellings (or ‘beading’) are a hallmark of axonopathy that has been documented since as early as Ramón y Cajal [33]. The causes of this phenomenon vary from toxins and genetic defects to inflammation and metabolic disturbances, e.g., insufficient autophagy [34–36]. In neurons, autophagy generally follows pathways not different from those described in yeast, yet with distinct spatial patterns [37, 38]. In axons, autophagosomes originate from distal growth cones, and have to be transported retrogradely towards the soma to fuse with lysosomes [39, 40]. In contrast, in the soma and dendrites, autophagosomes are formed locally and remain confined [41]. This is in line with our observation with the autophagy reporter assay, where acidized autophagy vacuoles could be robustly observed near or within the soma of wild-type iGlutNs, but not in FIP200^KO^ or MRT-treated neurons. We also show that blockage of autophagy initiation, either by FIP200 loss of function mutation or ULK1 inhibition, leads to pathological axonal swellings. This observation is in agreement with a previous report that axonal swellings could be observed in murine cerebellar neurons lacking FIP200 [14]. However, a recent study did not report significant axonal swelling induction in FIP200 knock-down neurons, but only in ATG5 or ATG16L1 knockout neurons in primary mouse neuronal cultures [42]. This divergent observation could possibly be due to an insufficient shRNA-mediated knockdown resulting in residual FIP200 activity. FIP200^KO^-induced autophagy deficiency also likely leads to a shift in proteostasis as well as homeostasis of metabolic and redox processes in induced human glutamatergic neurons. Despite the blockage of autophagy initiation, accumulation of p62, and initial axonopathy, no major difference in axonal or dendrite growth was evident in FIP200^KO^ iGlutNs in the present study. We also observed no accumulation of ubiquitin that would hint to decreased proteasome activity. This finding stands somewhat at odds with previous observations in transgenic mice with neural-specific FIP200 deletion, which showed accumulation of both p62 and ubiquitin in the cerebellum [14]. While we have no explanation for this incongruence, it could well be due to phenotypic differences between neuronal subtypes and species, with our model reflecting not mouse cerebellar neurons but forward-programmed human iGlutNs.

Interestingly, a FIP200^KO^ did not compromise the basal electrophysiological function of iGlutNs, but resulted in hyperactivity that can be attributed to stronger glutamatergic synaptic transmission. Data from MRT-treated cultures implicate autophagy deficiency to play a role in this phenomenon. Previous studies have reported that autophagy blockage could lead to a stronger overall synaptic transmission by regulating presynaptic vesicles [43], reducing spine pruning [44], or elevating calcium release from the endoplasmic reticulum [45]. While we did not observe a quantitative difference in synaptic proteins between FIP200^KO^ and wild-type neurons, the various other pathways were not addressed in the present study. Thus, future studies are required to further explore which mechanisms mediate autophagy deficiency-induced hyperactivity in our system. Our experimental observations also suggest that FIP200 may modulate neuronal activity through FAK signaling pathways. FAK regulates dendrite arborization under the control of protocadherins [46–48]. Reduced expression of protocadherins and deficits in arborization and synapses were recently reported in iPSC-derived cortical interneurons from schizophrenia patients, and these phenotypes could be rescued by inhibitors of FAK pathways [49]. As loss of function of FIP200 could lead to disinhibition of FAK [7], this could explain the downregulation of PTPRT and dysregulation of cell adhesion-related processes, which are crucial for morphological development, neurite outgrowth, migration, and other neuronal properties [50, 51]. Since we did not observe a difference in neurite outgrowth between FIP200^KO^ and control neurons, the successful rescue of hyperactivity by the presumed FAK ‘re-inhibition’ in FIP200^KO^ neurons might be based on other mechanisms. Further studies focusing on cell adhesion-related processes, especially FAK and PTPRT signaling pathways, will be required to address this question. Besides, FAK can modulate AKT-mTOR signaling in multiple ways [52–54], thus linking the focal adhesion processes with metabolic regulation and autophagy. Therefore, future studies addressing the interplay of FAK-related pathways and autophagy in the context of neuronal function would be of particular interest.

Some limitations of the present study remain to be addressed. For instance, all experiments were performed using cryopreserved neurons. While this approach largely facilitates experimental standardization across different batches, we cannot entirely rule out that cryopreservation might enhance the observed pathophenotypes. In addition, NGN2-induced neuronal populations may include smaller fractions of other cell types, such as GABAergic and peripheral neurons as well as non-neuronal cells [55]. Although our quality control studies in the context of this and other projects indicate that the level of heterogeneity is comparable across different genotypes, it can, in principle, impact neuronal network activity. Another bias is the co-culture with astrocytes, which is required for optimal synaptic maturation, but can lead to lower specificity and statistical power of the proteomic analyses due to the presence of both mouse and human polypeptides. Furthermore, although PF228 is a well-established and potent FAK inhibitor [32, 56], future studies should ideally include a direct assessment of FAK activity in FIP200^KO^ iGlutNs and subsequent (re-)inhibition of FAK by PF228. It is also fair to note that most pathophenotypes reported in this study were detected in still young yet synaptically active neurons. Long-term in vitro studies and potentially in vivo transplantation may provide a more comprehensive understanding of the functions of FIP200 in fully mature, adult-like human neurons.

Human brain imaging studies consistently reported hippocampal and cortical hyperactivity in schizophrenia patients [57–60]. Several mechanisms have been proposed regarding the origin of hippocampal hyperactivity, e.g., glutamate [61], GABA [57, 60], or dopamine dysregulation [62]. Our observations suggest that loss of function of FIP200 mediates hyperactivity in glutamatergic neurons, which in turn is implicated in schizophrenia symptoms. However, the specific roles of FIP200 in hPSC-derived glutamatergic neurons need to be further investigated. Specifically, it remains to be determined which mechanisms mediate the strengthened glutamatergic activation in FIP200^KO^ neurons and how these to-be-defined mechanisms could be linked to autophagy or possibly FAK pathways. It is also unclear whether a gain of function or a reduction/loss of function of FIP200 could promote the development of schizophrenia and other neuropsychiatric conditions, either of which could be pathogenic. Therefore, future work with patient-specific mutations of FIP200 is needed to obtain a clearer picture.

## Supplementary information

Supplementary Figure S1

Supplementary Figure S2

Supplementary Figure S3

Supplementary Figure S4

Supplementary Figure S5

Supplementary Figure S6

Supplementary Figure S7

Supplementary Figure S8

Supplementary Table S1

Supplementary Table S2

Supplementary Table S3

Supplementary Table S4

## Data Availability

The proteomics raw data, including details on the LC-MS/MS parameters, have been uploaded to the ProteomeXchange Consortium via the MassIVE partner repository (https://massive.ucsd.edu) with the dataset identifier MSV000091643.
