# Supplementary material for: Loss of function of FIP200 in human pluripotent stem cell-derived neurons leads to axonal pathology and hyperactivity
Source: Transl Psychiatry. 2023 May 3;13:143. doi: 10.1038/s41398-023-02432-3 (PMC10156752; doi:10.1038/s41398-023-02432-3)
Supplement: Supplementary file 1 — Supplementary Figure S1 [file 41398_2023_2432_MOESM1_ESM.pdf]

Figure S1.

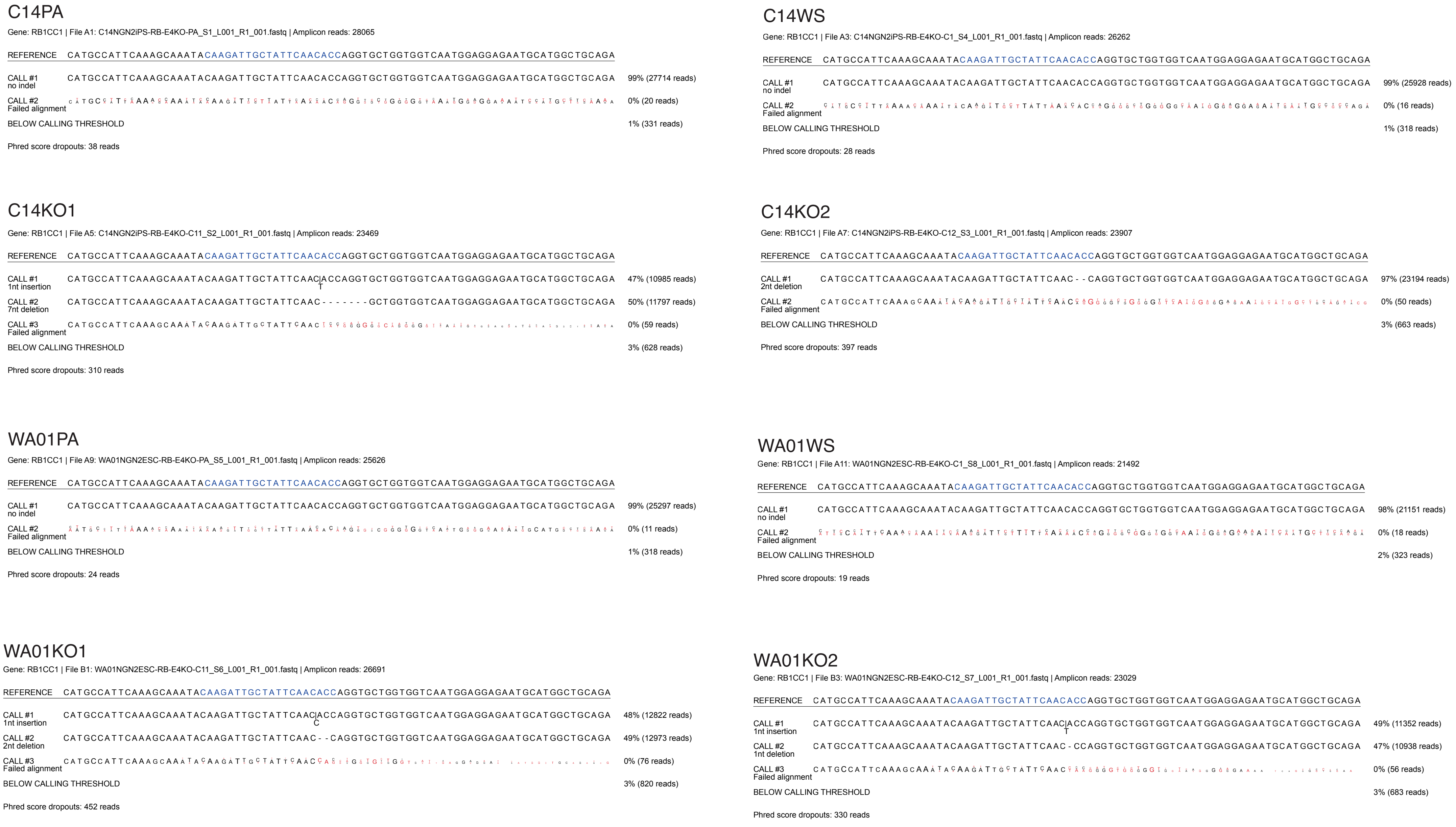

Figure S1. FIP200 genotype validation via deep sequencing. Shown are amplicon sequencing results from the FIP200 target region of individual candidate clones. Blue letters in the reference sequence mark the gRNA target sequence.
