# Supplementary material for: Loss of function of FIP200 in human pluripotent stem cell-derived neurons leads to axonal pathology and hyperactivity
Source: Transl Psychiatry. 2023 May 3;13:143. doi: 10.1038/s41398-023-02432-3 (PMC10156752; doi:10.1038/s41398-023-02432-3)
Supplement: Supplementary file 2 — Supplementary Figure S2 [file 41398_2023_2432_MOESM2_ESM.pdf]

Figure S2.

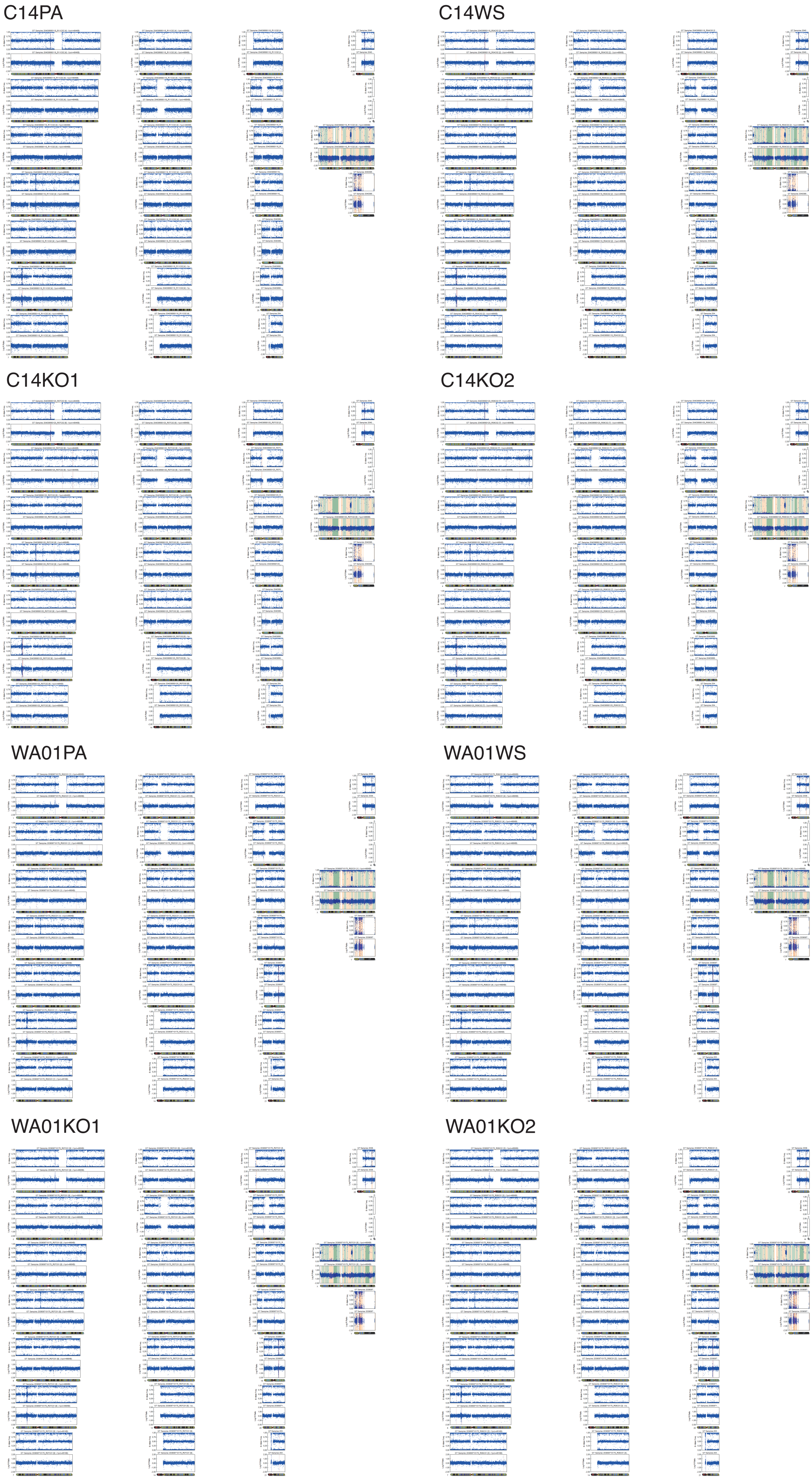

Figure S2. Genetic integrity of all hPSC lines used in this study was assessed by SNP genotyping. For each chromosome the B allele frequency (upper row) and the log R ratio (lower row) are shown. No major genomic alterations could be detected.
