# Supplementary material for: Loss of function of FIP200 in human pluripotent stem cell-derived neurons leads to axonal pathology and hyperactivity
Source: Transl Psychiatry. 2023 May 3;13:143. doi: 10.1038/s41398-023-02432-3 (PMC10156752; doi:10.1038/s41398-023-02432-3)
Supplement: Supplementary file 3 — Supplementary Figure S3 [file 41398_2023_2432_MOESM3_ESM.pdf]

Figure S3

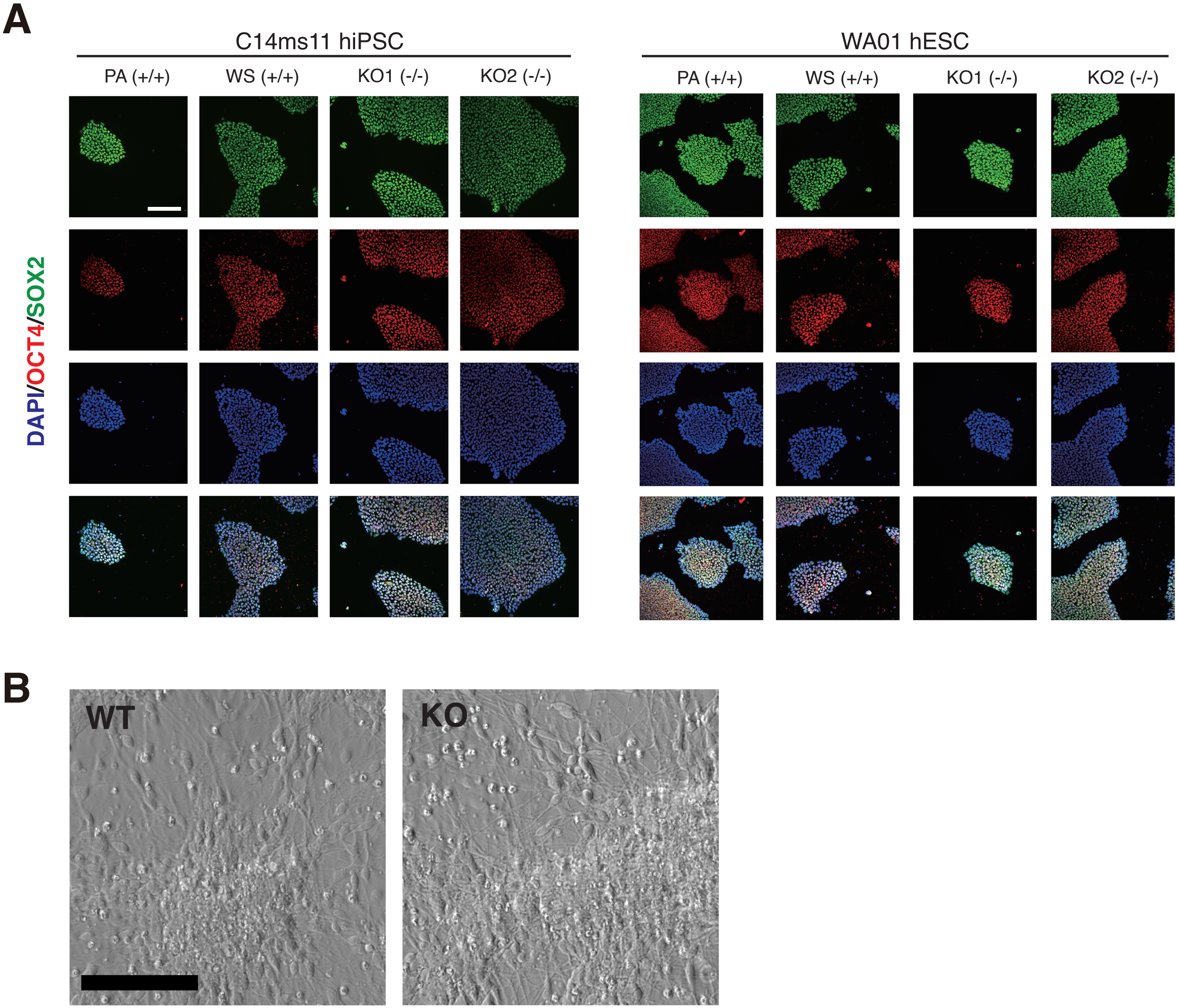

Figure S3. Quality control of iPSCs and forward programmed neurons. (A) Immunocytochemistry of the indicated hPSC lines for detection of the pluripotency markers OCT4 and SOX2. PA, parental line; WS, wild type subclone; KO, knock out line. (B) Example bright field pictures of forward-programmed neurons derived from FIP200^{KO} and wild-type cultures before cryopreservation at Day 8 of neuronal induction (timepoint shown in Figure 1C). Scale bars, 100 μm.
