# Supplementary material for: Loss of function of FIP200 in human pluripotent stem cell-derived neurons leads to axonal pathology and hyperactivity
Source: Transl Psychiatry. 2023 May 3;13:143. doi: 10.1038/s41398-023-02432-3 (PMC10156752; doi:10.1038/s41398-023-02432-3)
Supplement: Supplementary file 4 — Supplementary Figure S4 [file 41398_2023_2432_MOESM4_ESM.pdf]

Figure S4

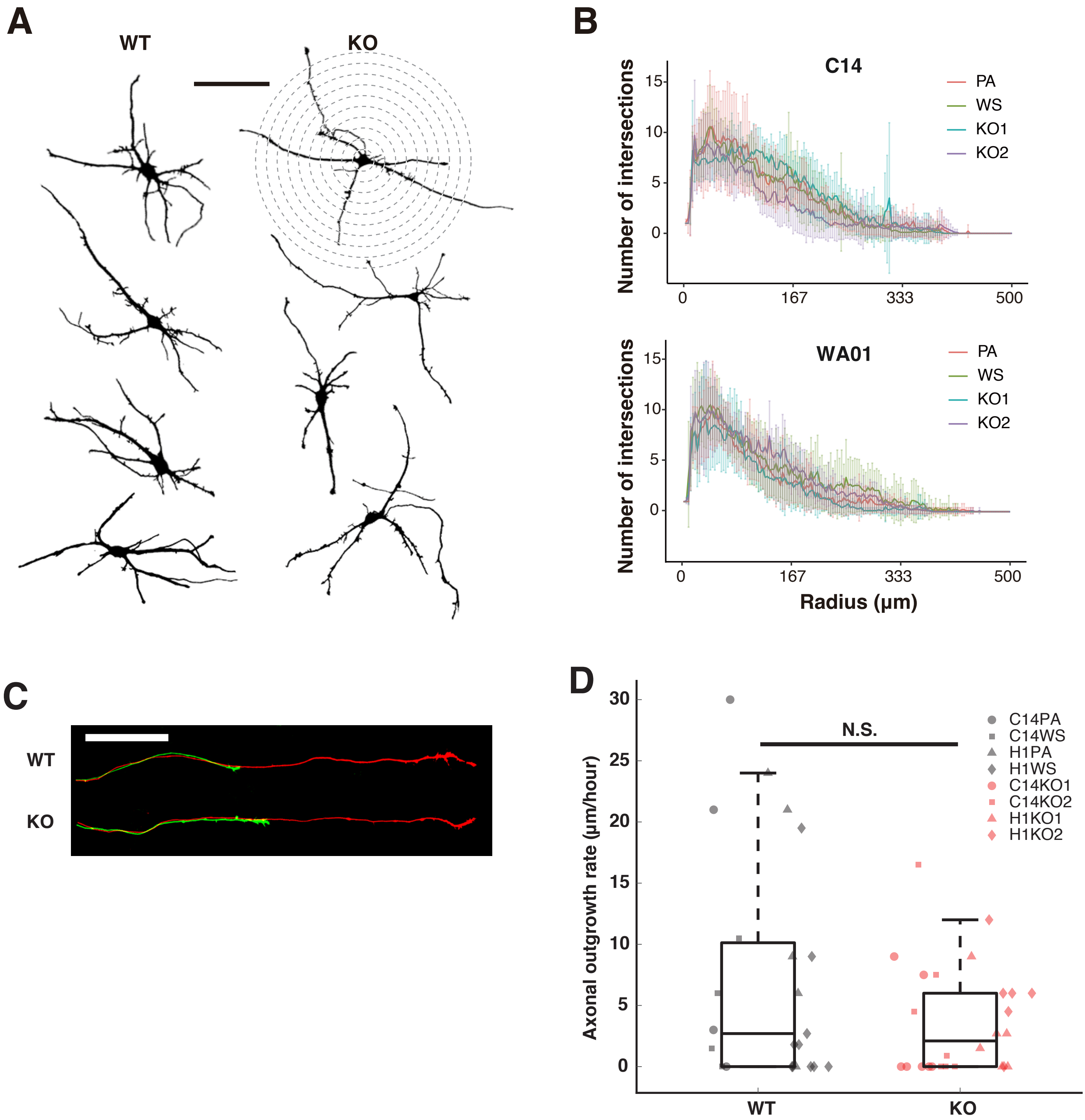

Figure S4 Morphological assessment of FIP200^{KO} and control iGlutNs. (A) Representative images of neurons in control and FIP200^{KO} iGlutN cultures. At the top right, a grid of concentric shells used for Sholl analysis is shown. Scale bar, 100 µm. (B) Sholl analysis results showing the number of dendritic intersections at fixed distances from the soma in concentric circles. For each cell line >10 pictures from 2 independent experiments were analyzed. (C) Example live fluorescence images showing axon growth in human iGlutNs. The initial positions (green) and the axons grown for 10 hours (red) are superimposed. Scale bar, 50 µm. (D) Quantification of axon outgrowth rate. The speed was calculated by dividing the difference in axon length in two images by the time interval. Analyses were performed during week 4 of maturation on mouse astrocytes.
