# Supplementary material for: Loss of function of FIP200 in human pluripotent stem cell-derived neurons leads to axonal pathology and hyperactivity
Source: Transl Psychiatry. 2023 May 3;13:143. doi: 10.1038/s41398-023-02432-3 (PMC10156752; doi:10.1038/s41398-023-02432-3)
Supplement: Supplementary file 5 — Supplementary Figure S5 [file 41398_2023_2432_MOESM5_ESM.pdf]

Figure S5

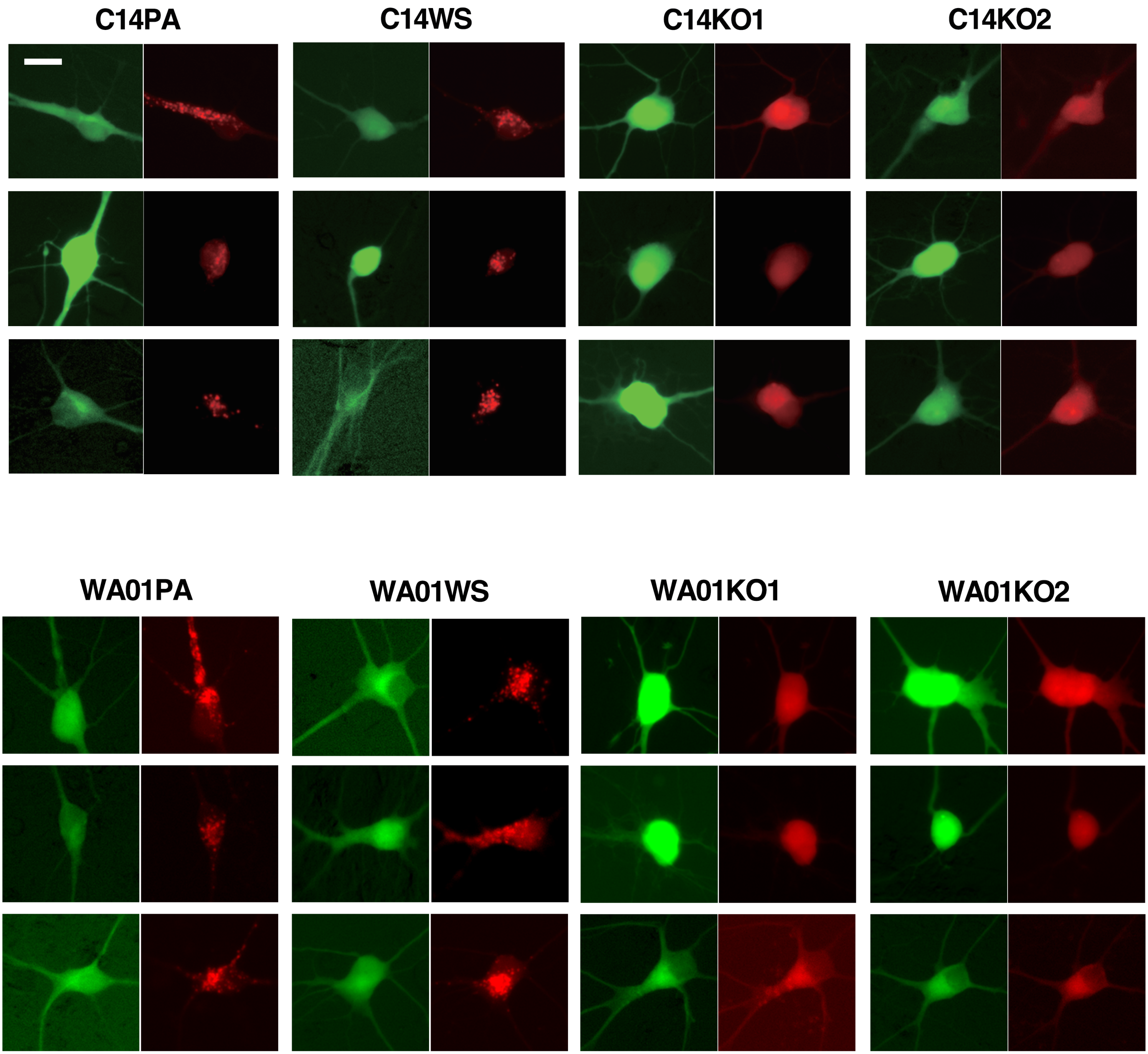

Figure S5 Representative exampe fluorescence images of iGlutN cultures transfected with the autophagy reporter ptfLC3. The first example of each group is also shown in Figure 2A, but with merged channels. Scale bar, 30µm.
