# Supplementary material for: Loss of function of FIP200 in human pluripotent stem cell-derived neurons leads to axonal pathology and hyperactivity
Source: Transl Psychiatry. 2023 May 3;13:143. doi: 10.1038/s41398-023-02432-3 (PMC10156752; doi:10.1038/s41398-023-02432-3)
Supplement: Supplementary file 6 — Supplementary Figure S6 [file 41398_2023_2432_MOESM6_ESM.pdf]

Figure S6

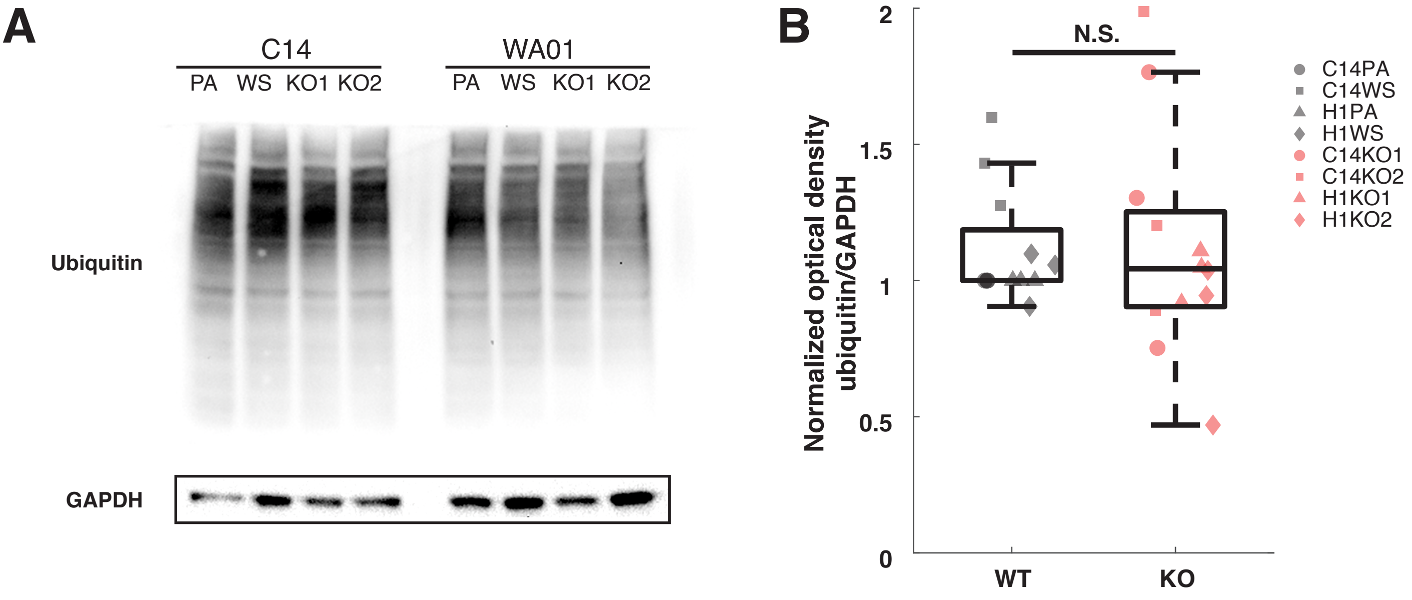

Figure S6. Assessment of proteasomal function in FIP200^{KO} and control iGlutNs. Qualitative (A) and densitometric (B) western blot analyses of ubiquitin (n = 3 independent experiments). Data were collected during week 4 of maturation.
