# Supplementary material for: Loss of function of FIP200 in human pluripotent stem cell-derived neurons leads to axonal pathology and hyperactivity
Source: Transl Psychiatry. 2023 May 3;13:143. doi: 10.1038/s41398-023-02432-3 (PMC10156752; doi:10.1038/s41398-023-02432-3)
Supplement: Supplementary file 7 — Supplementary Figure S7 [file 41398_2023_2432_MOESM7_ESM.pdf]

Figure S7

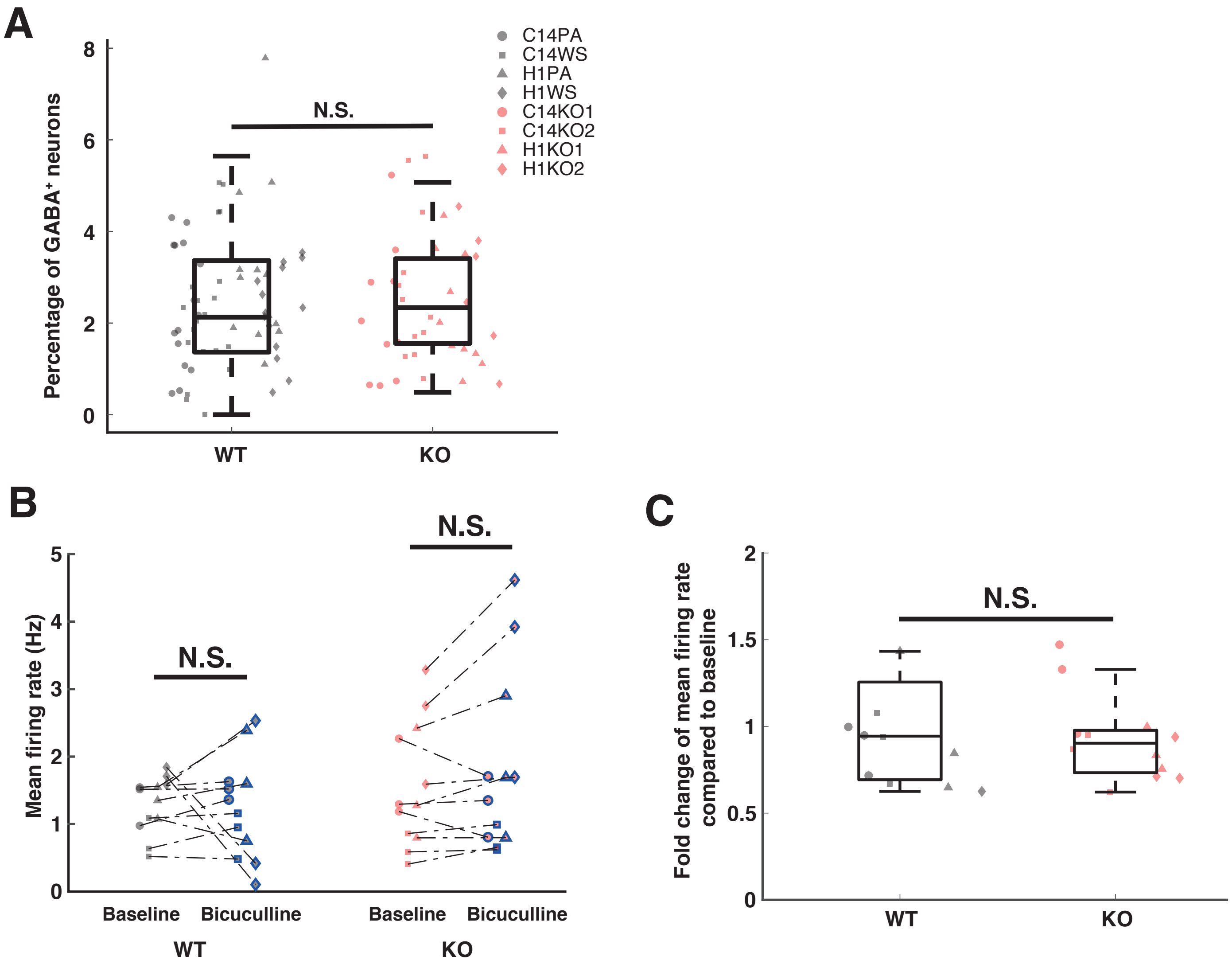

Figure S7. Contribution of GABAergic neurons to spiking activity of wild type and FIP200^{KO} iGlutN cultures. (A) Quantification of GABA positive neurons in wild type and FIP200^{KO} iGlutN cultures. (B-C) Electrophysiological data relating to the quantification of (B) mean firing rate in FIP200^{KO} and control iGlutN cultures before and after treatment with 10 µM bicuculline, and (C) the fold change of mean firing rate. All data were acquired during week 4 of maturation on mouse astrocytes.
