# Supplementary material for: Loss of function of FIP200 in human pluripotent stem cell-derived neurons leads to axonal pathology and hyperactivity
Source: Transl Psychiatry. 2023 May 3;13:143. doi: 10.1038/s41398-023-02432-3 (PMC10156752; doi:10.1038/s41398-023-02432-3)
Supplement: Supplementary file 8 — Supplementary Figure S8 [file 41398_2023_2432_MOESM8_ESM.pdf]

Figure S8

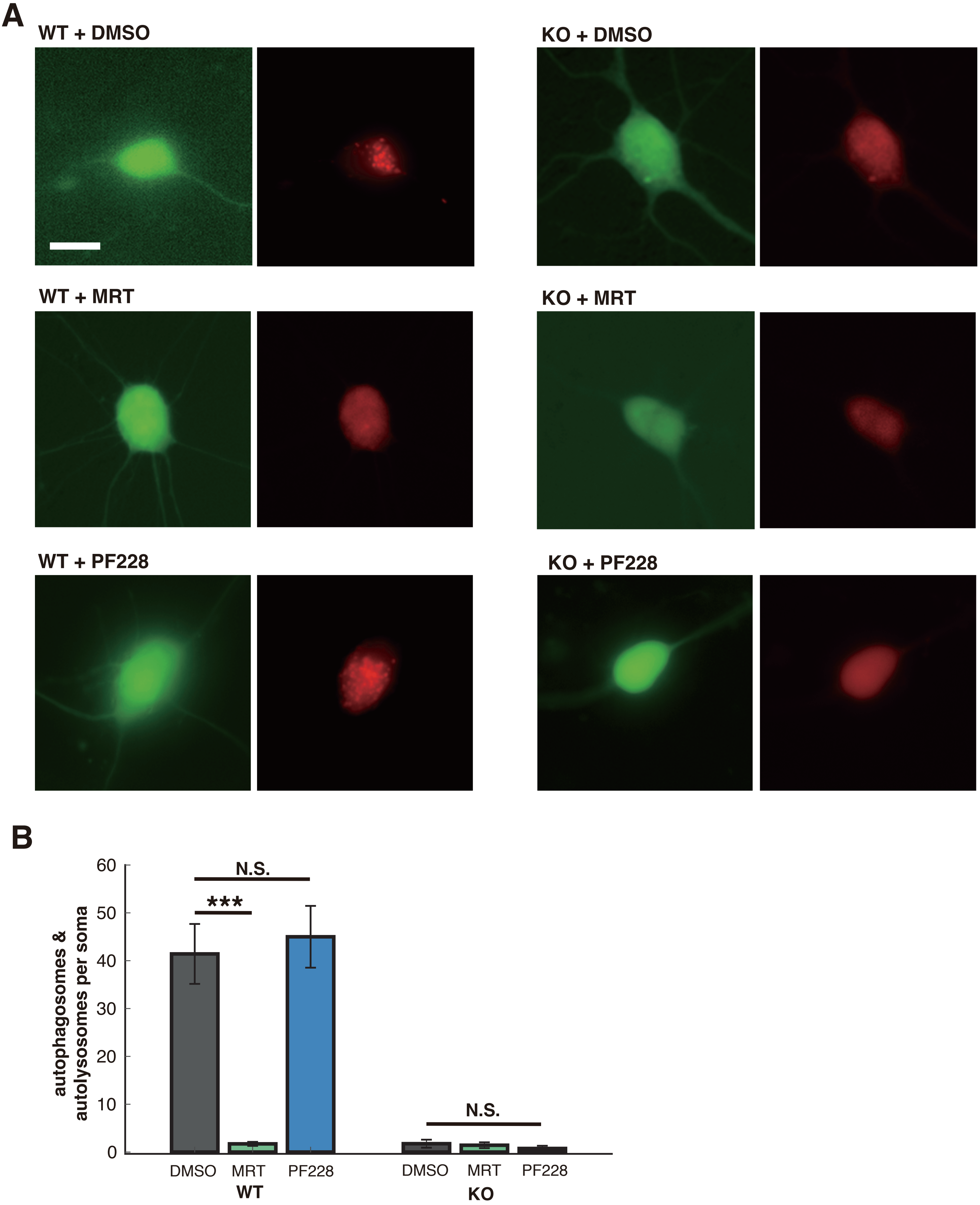

Figure S8. The effect of MRT (ULK1/2 inhibitor) or PF228 (FAK inhibitor) on autophagy in wild type and $FIP200^{KO}$ iGlutN cultures. (A) Representative live fluorescence microscopy pictures of iGlutN cultures treated with 3.5 µM MRT or 1 µM PF228. Scale bar, 30µm. (B) Quantification of autophagosomes and autolysosomes in wild type and $FIP200^{KO}$ iGlutNs treated with MRT or PF228. 3 pictures from each of the eight cell lines in each condition were analyzed.
