# Supplementary material for: Loss of function of FIP200 in human pluripotent stem cell-derived neurons leads to axonal pathology and hyperactivity
Source: Transl Psychiatry. 2023 May 3;13:143. doi: 10.1038/s41398-023-02432-3 (PMC10156752; doi:10.1038/s41398-023-02432-3)
Supplement: Supplementary file 9 — Supplementary Table S1 [file 41398_2023_2432_MOESM9_ESM.docx]

**Table S1.** Isogenic hPSC clones with FIP200 loss of function mutations used in the present study.

| **Background** | **Genotype** | **Cell line** | **FIP200 KO mutations*** |
| --- | --- | --- | --- |
| **C14** (iLB-C14m-s11; <https://hpscreg.eu/cell-line/UKBi017-A>)  Generated from skin fibroblasts of a healthy Caucasian male. | Wild type | C14-PA | N.A. |
|  |  | C14-WS | N.A. |
|  | FIP200^KO^ | C14KO1 | c.124_125insT/c.125_131del |
|  |  | C14KO2 | c.125_126del/c.125_126del |
| **WA01** (WAe001-A; <https://www.wicell.org/home/stem-cells/catalog-of-stem-cell-lines/wa01.cmsx>)  Obtained from WiCell Research Institute. | Wild type | WA01-PA | N.A. |
|  |  | WA01-WS | N.A. |
|  | FIP200^KO^ | WA01-KO1 | c.124_125insC/c.125_126del |
|  |  | WA01-KO2 | c.124_125insT/c.125del |

* Referring to FIP200 (RB1CC1) CCDS34892.1.
